# Supplementary material for: A Colorimetric Assay for the Detection of Glucose and H2O2 Based on Cu-Ag/g-C3N4/ZIF Hybrids with Superior Peroxidase Mimetic Activity
Source: Molecules. 2020 Sep 27;25(19):4432. doi: 10.3390/molecules25194432 (PMC7583779; doi:10.3390/molecules25194432)
Supplement: Supplementary file 1 [file molecules-25-04432-s001.pdf]

Supporting Information

A Colorimetric Assay for the Detection of Glucose and H_2O_2 Based on Cu-Ag/g-C_3N_4/ZIF Hybrids with Superior Peroxidase Mimetic Activity

Quan Pan [1,†], Yuelin Kong [2,†], Kuan Chen [2], Mi Mao [2], Xiaohui Wan [1], Xiaoyan She [1], Qingsong Gao [1], Yu He [2,*] and Gongwu Song [2]

1 Hubei Province Fiber Inspection Bureau, Wuhan 430061, China; panquanfq@163.com (Q.P.); yangguangwxh9272@163.com (X.W.); sxy13871171608@163.com (X.S.); gqs0601@163.com (Q.G.)

2 Hubei Collaborative Innovation Center for Advanced Organic Chemical Materials, Ministry-of-Education Key Laboratory for the Synthesis and Application of Organic Functional Molecules, College of Chemistry and Chemical Engineering, Hubei University, Wuhan 430062, China; kongyue_lin@163.com (Y.K.); workout_k@163.com (K.C.); xxhh_my@163.com (M.M.); songgw@hubu.edu.cn (G.S.) heyu@hubu.edu.cn (Y.H)

* Correspondence: heyu@hubu.edu.cn

† These authors contributed equally to this work.

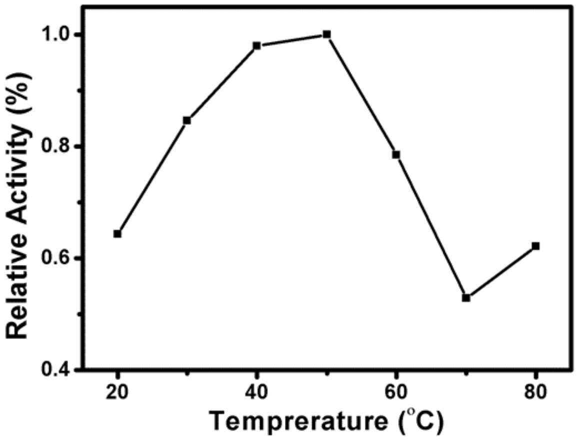

Figure 1. Effects of different temperatures on catalytic activity of Cu-Ag/g-C_3N_4/ZIF.

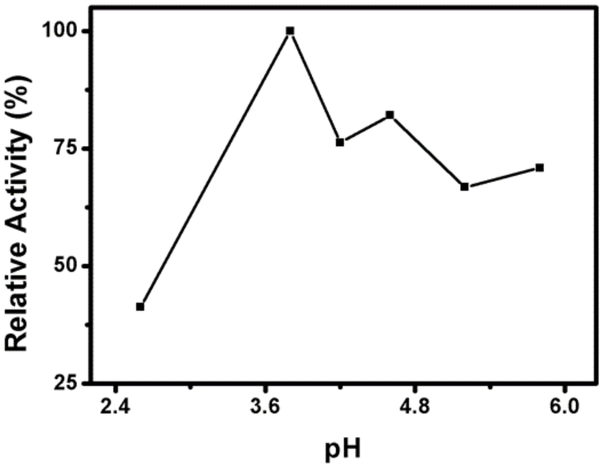

Figure 2. Effects of different pH on catalytic activity of Cu-Ag/g-C_3N_4/ZIF.

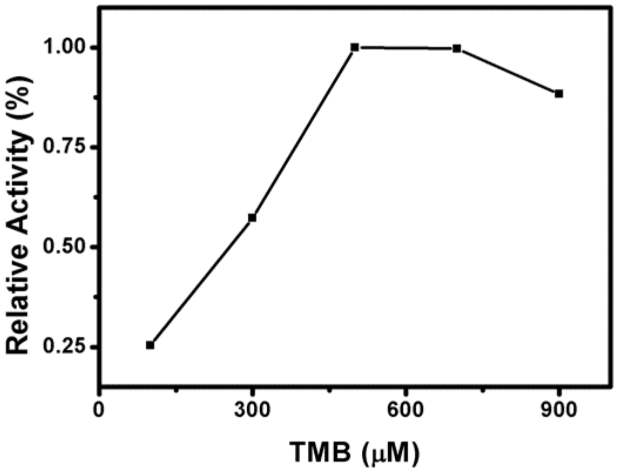

Figure 3. Effects of different concentration of TMB on catalytic activity of Cu-Ag/g-C_3N_4/ZIF.
